# Depressive Symptoms and PANSS Symptom Dimensions in Patients With Predominant Negative Symptom Schizophrenia: A Network Analysis

**DOI:** 10.3389/fpsyt.2022.795866

**Published:** 2022-04-25

**Authors:** Koen Demyttenaere, Elizabeth Anthonis, Károly Acsai, Christoph U. Correll

**Affiliations:** ^1^Psychiatry Research Group, Department of Neurosciences, Faculty of Medicine, University of Leuven, and University Psychiatric Center KU Leuven, Leuven, Belgium; ^2^University Psychiatric Center KU Leuven, Leuven, Belgium; ^3^Ceva Animal Health, Ceva-Phylaxia, Budapest, Hungary; ^4^Department of Psychiatry, The Zucker Hillside Hospital, Northwell Health, Glen Oaks, NY, United States; ^5^Department of Psychiatry and Molecular Medicine, Donald and Barbara Zucker School of Medicine at Hofstra/Northwell, Hempstead, NY, United States; ^6^Department of Child and Adolescent Psychiatry, Charité Universitätsmedizin Berlin, Berlin, Germany

**Keywords:** cariprazine, risperidone, schizophrenia, negative symptoms, Positive and Negative Syndrome Scale (PANSS), Calgary Depression Scale for Schizophrenia (CDSS)

## Abstract

**Introduction:**

Schizophrenia is a severe psychiatric disorder with a large symptomatic heterogeneity. Moreover, many patients with schizophrenia present with comorbid psychiatric symptoms or disorders. The relation between depressive symptoms and negative symptoms, such as blunted affect, alogia, anhedonia, asociality and avolition, is particularly intriguing. The negative symptoms can be primary or secondary of depression or overlapping with depressive symptoms. The aim of the present network analysis was to better understand the interactions between depressive symptoms and the different symptoms of schizophrenia and to investigate whether negative symptoms and depressive symptoms can be better delineated.

**Methods:**

A network analysis on the baseline item scores of the Positive and Negative Syndrome Scale (PANSS) and Calgary Depression Scale for Schizophrenia (CDSS) from the cariprazine-risperidone study in patients with predominant negative symptoms (PNS) was performed. The connections between all these symptoms (PANSS and CDSS) were investiged: node strength and network centrality were estimated and the Mohr 5-factor model of the PANSS was applied to test the validity of its different symptoms clusters.

**Results:**

Across 460 patients with schizophrenia and PNS, the most central symptom (largest node strength) was depression (PANSS) followed by depression (CDSS), anxiety, lack of judgment and insight and tension. The PANSS negative symptom cluster together and was only poorly connected with CDSS depresson symptoms. The Mohr 5 factor model was clearly recognized in the overall clustering of symptoms.

**Conclusion:**

This network analysis suggests that depression and anxiety symptoms are the most central in this PNS patient population, despite the baseline low depression scores, and that negative symptoms are a clearly independent symptom cluster that can be delineated from depressive symptoms.

## Introduction

Schizophrenia is a severe mental disorder with heterogeneous symptom constellations involving cognitive, behavioral and emotional symptoms but with no single symptom being pathognomonic of the disorder (1, 2). How to best address this heterogeneity or how to best rank the importance of individual symptoms has been cause of much debate and much confusion in the literature, as well regarding the classification systems as regarding the assessment tools. Moreover, many patients with schizophrenia present with comorbid psychiatric symptoms or disorders: an increased prevalence of anxiety, depressive and substance use disorders has been documented (3) and comorbidity results in higher suicidality rates (4). Schizophrenia without psychiatric comorbidity has also been shown to be associated with better overall mental health but also with poorer illness and treatment insight compared to those patients with anxious and depressive disorder comorbidity (5). Depressive symptoms as well as full mood episodes are common in schizophrenia but should be present for only a relatively brief period (hence differentiating schizophrenia from schizoaffective disorder). Depressive symptoms show a modal prevalence of about 25% and they may occur in all phases of schizophrenia (3).

Regarding diagnostic criteria, the 5^th^ edition of the Diagnostic Manual of Mental Disorders (DSM-5), in an attempt to better delineate different symptom clusters in schizophrenia, dropped the subtyping approach and chose for a dimensional approach suggesting a severity rating based on a quantitative assessment of the 5 primary symptom domains of psychosis including delusions, hallucinations, disorganized speech, abnormal psychomotor behavior, and negative symptoms while adding that the assessment of 3 other domains (cognition, depression and mania symptom domains) is also vital (2). The importance of depressive symptoms is therefore explicitly recognized. The understanding of the relation between these different domains is still suboptimal.

Regarding the assessment tools, the original Positive and Negative Syndrome Scale (PANSS) comprised a positive symptom subscore, a negative symptom subscore and a general psychopathology subscore (the latter including items such as guilt feelings, depression, poor attention and motor retardation all referring to standard depressive disorders). Later, **several other dimensional models** (based on factor analysis) of the PANSS have been suggested. The 5-factor structure, which generally includes positive, negative, cognitive/disorganization, depression/anxiety, and excitability/hostility domains, is the basis of most models, including the Lindenmayer, Marder, Mohr and the latest Wallwork models. Although several factor analyses studies have suggested that a 5-factor model captures PANSS structure better than the original PANSS subscales, no single model has achieved broad consensus, and the 3 original subscales are still widely used. In a recent paper, a network analysis illustrated the validity of the Mohr model (6).

Regarding the depressive symptoms in the PANSS, the anhedonia symptom which is a core depressive symptom is remarkably absent in the PANSS and therefore also in the more recently proposed 5-factor models like in the Mohr depression/anxiety factor of the PANSS (which has 5 items: G1 being somatic concern; G2 being anxiety, G3 being guilt feelings, G4 being tension and G6 being depression). A more recently developed depression scale for patients with schizophrenia [the Calgary Depression Rating Scale for Schizophrenia (CDSS) (3, 7, 8)] also omits anhedonia (as well as the psychomotor symptoms retardation/agitation) as depressive symptom. The reason for these omissions could well be their possible overlap with other schizophrenia symptoms: anhedonia can be a depressive symptom or a schizophrenia negative symptom, psychomotor retardation can be a depressive symptom or a schizophrenia negative symptom (withdrawal), psychomotor agitation can be a depressive symptom or a schizophrenia excitement/hostility symptom. On the other hand, suicidality as a depressive symptom is included in the CDSS but absent in the PANSS (8, 9). Again, the link between depressive symptoms and the other schizophrenia symptom domains (especially the positive symptoms, the primary negative symptoms, the cognitive symptoms and the depression/anxiety symptoms) or the differentiation between depressive symptoms and antipsychotic side effects (including dysphoria, akinesia and akathisia) are insufficiently understood (10).

The relation between depressive symptoms and negative symptoms is particularly intriguing. **Negative symptoms** (11) such as blunted affect, alogia, anhedonia, asociality and avolition can indeed be primary or secondary and it is widely believed that most of the currently available treatments are more efficacious on secondary than on primary negative symptoms (12). Secondary negative symptoms can be the consequence of positive symptoms (withdrawing because of persecutory delusions, or withdrawing as a coping strategy when feeling unable to process overwhelming external stimuli associated with psychotic experiences), or of cognitive symptoms (avolition and withdrawal because of impaired executive function or impaired retrieval of information), or of antipsychotic medication (side effects), or of environmental deprivation (social isolation and hospitalization). The negative symptoms can also be secondary of depression or overlapping with depressive symptoms (13). The relation between negative symptoms and depressive symptoms is not fully understood.

Recently, network analyses have been introduced in psychiatry research in an attempt to better understand the relations and interactions between the symptoms of a given psychiatric disorder: here symptoms are seen as a network, or as a system of entities that have connections with each other and that can influence one another (14, 15). It allows for a new conceptualization of mental disorders where symptoms can be ranked according to their centrality (number and strength of connections with the other symptoms of the disorder) and where a visualization of the relations enables to see which symptoms are more or less closely related.

The present paper reports on a centrality network analysis that was performed on the PANSS and the CDSS items in patients with “predominantly negative symptoms” (PNS) of schizophrenia that were enrolled in a double-blind trial with cariprazine or risperidone, where treatment with cariprazine resulted in a greater reduction of negative symptoms that was statistically significant and clinically meaningful (16). The first aim of the analysis was to better understand the interactions between depressive symptoms and the different symptoms of schizophrenia and to investigate whether negative symptoms and depressive symptoms can be better delineated. The second aim was to investigate whether the validity of the Mohr 5-factor model (re-organizing the PANSS items) can be illustrated in a network analysis.

## Materials and Methods

### Participants

The analyses were performed on patient data (*N* = 460) from the 26 week randomized, double-blind trial with long-term (>2year), stable schizophrenia and predominant negative symptoms: i.e. patients needed to be to in a stable condition (i.e., no psychiatric hospital admissions, acute exacerbations, or imprisonments) for at least 6 months before screening and they were required to suffer from predominant negative symptoms for at least 6 months with a PANSS factor score for negative symptoms of 24 or more, and a score of 4 or more on at least two of three core negative PANSS items (blunted affect, passive or apathetic social withdrawal, lack of spontaneity and flow of conversation) at screening and during a lead-in period (17). To ensure that it were not secondary negative symptoms, patients with a PANSS factor score for positive symptoms of more than 19 or with a score or 4 or more on two or more positive PANSS items (delusions, hallucinatory behavior grandiosity, suspiciousness, or unusual thought content) were ineligible as were patients with moderate or severe depressive symptoms (CDSS total score > 6) or patients with clinically relevant parkinsonism (investigator judged or score > 3 on the sum of the first eight items of the Simpson-Angus Scale) (8, 9, 17). In order to maximize the amount of information, all patients with baseline values (even those without post-baseline data) were included in the performed network analyses in both groups. Since only one patient had a CDSS suicidality item score different from 0, this one item was not integrated in the analysis.

### Statistical Analysis

The network structure was estimated for all the items (30 items of the PANSS and 9 items of the CDSS). A network is a representation of a system of nodes that are connected in one way or another (14, 15). In a network analysis edges connect the different nodes. For this study the nodes were the different items of the PANSS and the CDDS and the edges were the partial correlation coefficients between the different items. Therefore, the relationship between items is represented by an edge after controlling for all the other connections in a network. A weighed undirected network was constructed by using the R package *qgraph*, according to the guidance form Epskamp et al. (18) where the strength of the correlation between two items was represented by the thickness of a connecting line. Controlling for false positive edges was done by using the least absolute shrinkage and selection operator (lasso), which was coupled with the extended Bayesian information criterion (EBIC) for model selection. This causes very small edges to be set to zero, therefore pushing them out of the network estimation. Every item's importance in the network was investigated using three measures, namely node strength (sum of all weighted connections), closeness (the multiplicative inverse of the sum of the length of the shortest paths between all other nodes and the node) and betweenness (number of times a node lies on the shortest path between two other nodes). The results were graphically represented with nodes that have stronger and/or more connections between each other being placed closer together.

Afterwards, node centrality was assessed based on node strength. Node centrality can be used to look at the structural importance of each node in a network (14, 15). Node strength was chosen as it stands for the direct influence of a node on the entire network.

Stability of the network was investigated by using the *bootnet* R package, by creating random subsamples with decreasing size from the whole population.

## Results

The baseline clinical and sociodemographic characteristics are given in Table 1 and describe this patient population with “persistent and predominantly negative symptoms.”

**Table 1:** Baseline sociodemographic and clinical characteristics of the study population (mean ± SD).

Age	40.5 ± 10.9
Duration of illness	12.5 ± 8.7
Sex, male/female (%)	57/43
PANSS-FSPS	8.7 ± 2.7
PANSS-FSNS	27.6 ± 2.5
PANSS-GPPFS	36.2 ± 5.5
CDSS	0.8 ± 1.3

*PANSS-FSPS, PANSS factor score for positive symptoms; PANSS-FSNS, PANSS factor score for negative symptoms; PANSS general psychopathology factor score; CDSS, Calgary Depression Scale for Schizophrenia*.

**Figure 1:**
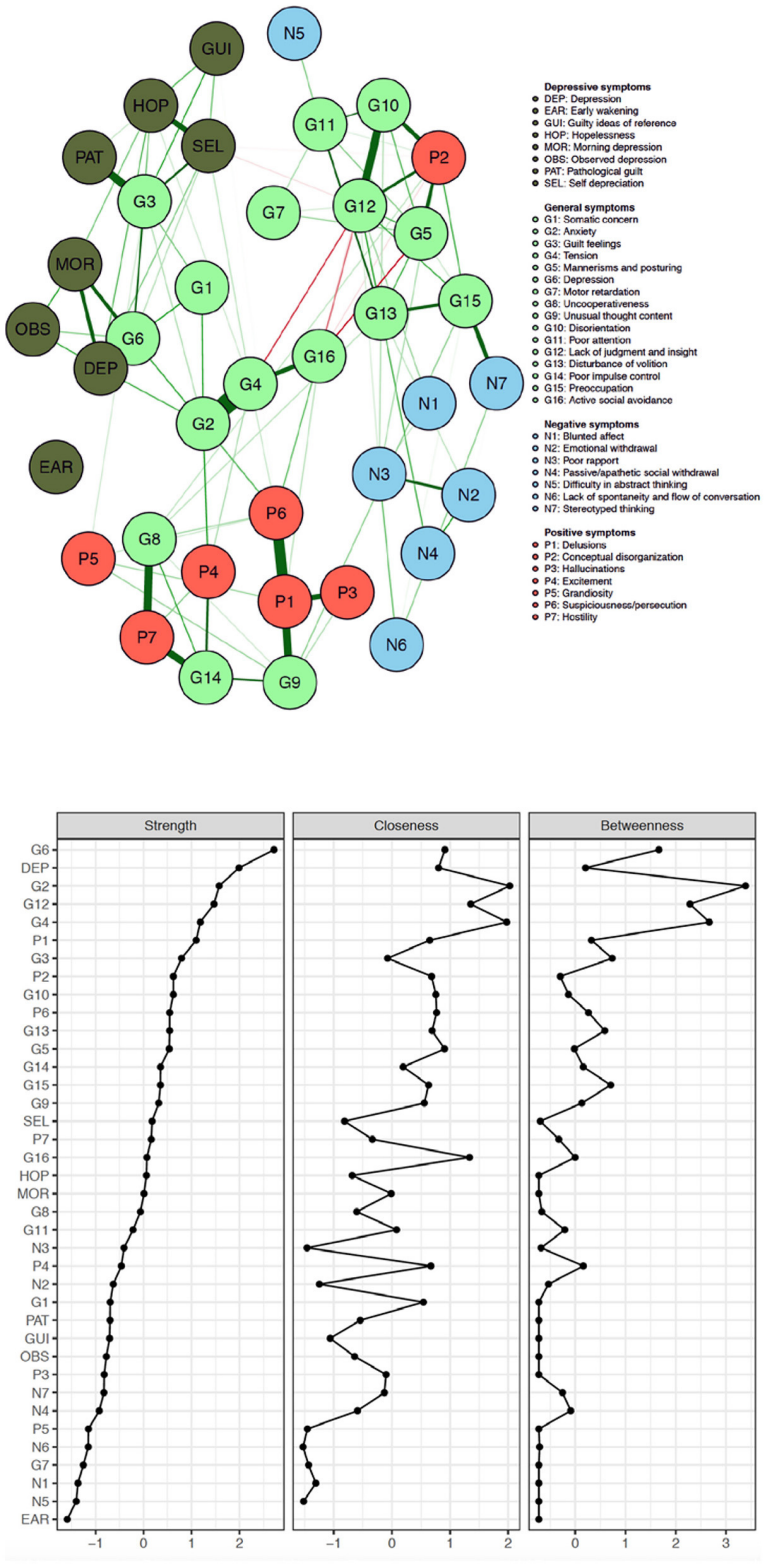
Network of PANSS symptoms and CDSS symptoms in patients with persistent and predominant negative symptoms (red, positive symptoms; blue, negative symptoms; pale green, general symptoms; dark green, CDSS symptoms).

**Figure 2:**
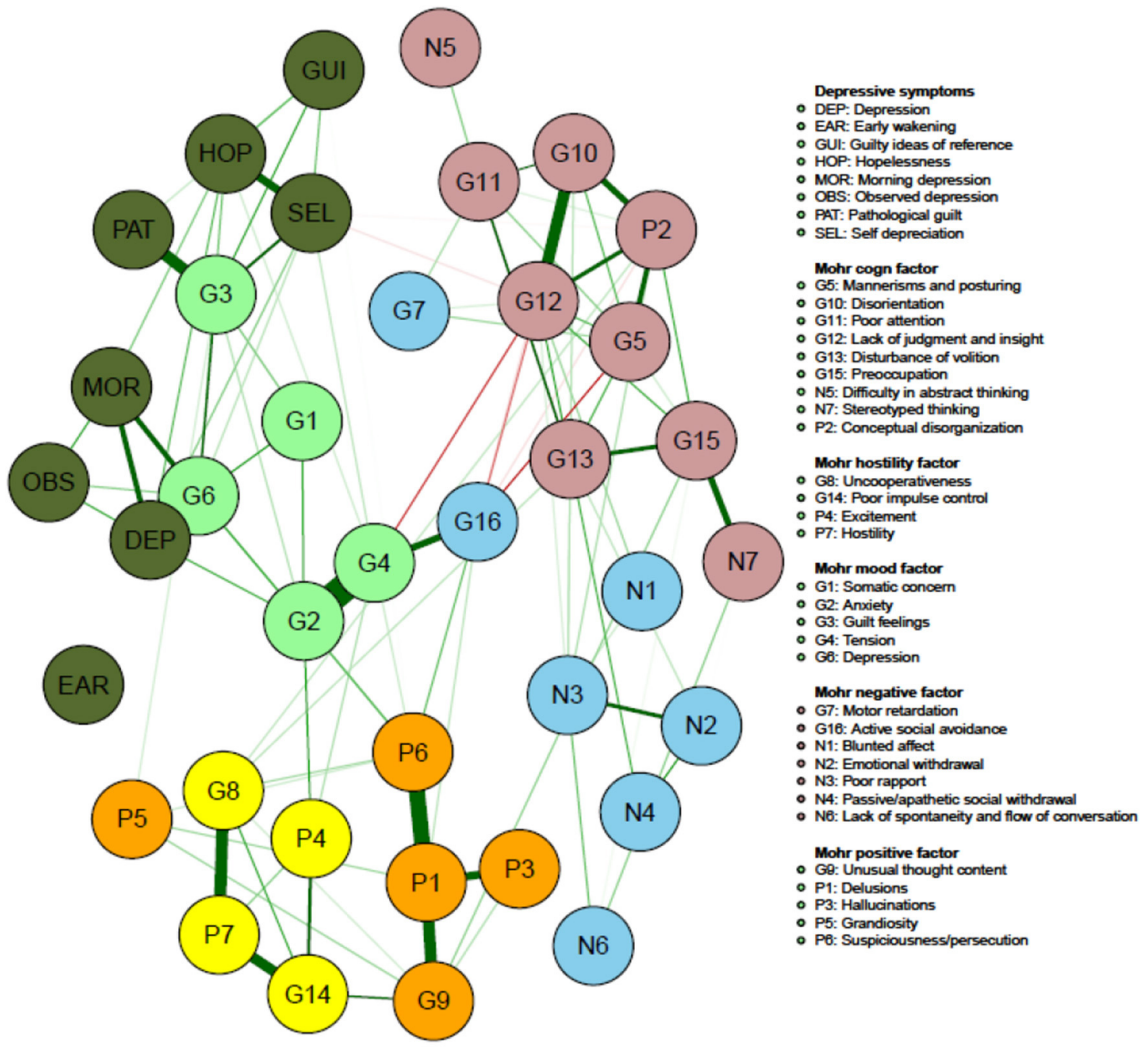
Network of PANSS symptoms and CDSS symptoms in patients with persistent and predominant negative symptoms (colors illustrating the Mohr 5-factor model of the PANSS; orange, positive symptom factor; yellow, hostility factor; blue, negative symptom factor; pink, cognitive symptom factor; pale green mood factor; dark green, CDSS symptoms).

Figure 1 visualizes the results of the network analysis of the individual PANSS and CDSS symptom items, while Figure 2 quantifies each of their ranking regarding node strength, closeness and betweenness.

The five items with the **largest node strength**, i.e., the items with the most frequent and the most intense connections with all the other CDSS and PANSS symptoms, are **depression (PANSS), depressed mood (CDSS), anxiety, lack of judgment/insight** and **tension**. The five items with the smallest node strength are lack of spontaneity and flow of conversation, motor retardation, blunted affect, difficulty abstract thinking and early wakening. The 5 negative symptoms according to the Mohr model all have a very low node strength: blunted affect (N1) comes in the 36^th^ position.

The original **PANSS negative symptoms** cluster closely together (except N5 –difficulty in abstract thinking) and all are poorly connected with other items and therefore have all a small node strength (ranking: N1 in 36^th^ position, N2 in 25^th^ position, N3 in 23^rd^ position, N4 in 32^nd^ position, N5 in 37^th^ position and N6 in 31^st^ position.

Figure 2 illustrates that the on factor analysis based Mohr 5 factor model of the PANSS is easily recognizable which is a kind of indirect validation.

Node strength is overall low for the **Mohr negative factor** (N1 being blunted affect; N2 being emotional withdrawal; N3 being poor rapport; N4 being passive/apathetic social withdrawal; N6 being lack of spontaneity and flow of conversation; G7 being motor retardation; G16 being active social withdrawal), with G16 having the highest strength in this group (but still only in the 16^th^ position on the ranking).

The **Mohr hostility/excitement symptoms** of the PANSS overall have an intermediate node strength (G8 being uncooperativeness; G14 being poor impulse control; P4 being excitement; P7 being hostility), G14 having the highest strength in this group.

Of the **Mohr positive symptoms**, P1 (delusions), P6 (suspiciousness/persecution) and G9 (unusual thought content) have a larger centrality than P3 (hallucinations) and P5 (grandiosity).

Regarding the **Mohr cognitive factor** (N5 being difficulty in abstract thinking; N7 being stereotyped thinking; G5 being mannerisms and posturing; G10 being disorientation; G11 being poor attention; G 12 being lack of judgment and insight; G 13 being disturbance of volition; G15 being preoccupation and P2 being conceptual disorganization), N5 and N7 show the lowest node strength.

Four (G2, G3, G4, G6) out of five items (G1 being somatic concern; G2 being anxiety; G3 being guilt feelings; G4 being tension; G6 being depression) of the **Mohr mood factor** show high node strength, with G6 showing the highest overall node strength.

## Discussion

An important finding of this network analysis is that four of the five items with the highest node strength were anxiety and depressive symptoms, which suggests that these had the most and the strongest connection with all the other symptoms. This is remarkable since this analysis was performed in a population of patients with schizophrenia with low baseline depression scores. This finding suggests that clinicians should probably, at least in a population with predominant negative symptoms, pay more attention to these symptoms and not only focus on the more socially disturbing positive symptoms and the more functionally impairing negative symptoms. It is hence understandable that the World Federation of Societies of Biological Psychiatry considers a “regular assessment of depressive symptoms” as mandatory for Good Clinical Practice (10). Depression indeed influences overall quality of life and life satisfaction. Moreover, subjective recovery in patients with schizophrenia is to a larger extend predicted by negative (depressive and anxious) emotion, self-esteem and hopelessness than by PANSS symptoms or by functioning (19). Depression is also related to suicidality: the lifetime risk of suicide and suicide attempt is patients with schizophrenia are 5 and 25–50%, respectively. A meta-analysis showed that amongst other variables depressive symptoms are higher in patients with suicide ideation, and that history of depression and depressive symptoms are associated with suicide attempts and that hopelessness is associated with suicide (20).

When looking at the negative symptoms, all except one (N5 difficulty in abstract thinking) cluster together and are poorly connected with other symptoms (apart from N7 stereotyped thinking and G15 preoccupation, which can be all be considered as cognitive symptoms). In a population with predominantly negative symptoms, the negative symptoms appear to be well distinguishable from depressive symptoms.

This partially contradicts previously published data suggesting that depressive and negative symptoms considerably overlap and that it is hence difficult to differentiate between both (21). The association between negative symptoms and depressive symptoms has not been sufficiently investigated and results are inconsistent (22). It should be remembered that in patients with schizophrenia negative symptoms can be primary or secondary and while in our selected population the negative symptoms are primary (6). This problem of differentiation between negative symptoms and depressive symptoms was already suggested in DSM-IV where it was stated that negative symptoms are difficult to evaluate and that a more phenomenological understanding can be helpful: depressive symptoms are considered to be associated with intense painful affect while negative symptoms are associated with diminution of affect and emptiness (23). Other studies suggested that low mood, suicidal ideation and pessimism have more specificity for depression while alogia and blunted affect may have more specificity as negative symptoms and while anhedonia, anergia and avolition may be common to both (24). Along the same lines, it has also been suggested that blunted affect (in the sense of inappropriate affect is a symptom of schizophrenia while decreased sponteaneous movements are regarded as unspecific and more relevant to the assessment of depression (11). As it has been suggested that anticipatory anhedonia is present in depression while consummatory anhedonia is present as well in depression as in schizophrenia (11). Another approach to differentiate is comparing depressive symptoms in depression with depressive symptoms in schizophrenia: it has been suggested that sleep disturbances and guilty ideas of reference are more typical for depression in patients with schizophrenia (25). The presently investigated patient population (stabilized with predominant negative symptoms, and without depression) makes it probably easier to differentiate between negative symptoms and depressive symptoms: one could assume that in other populations, the differentiation between primary negative symptoms and secondary negative symptoms (e.g., secondary to depression) could be more difficult and that a network analysis could well show a different constellation in a population with acute exacerbation of schizophrenia.

Another key question is of course whether the assessment of depressive and negative symptoms with the CDSS and with the PANSS depression/anxiety subscale are satisfactory. Anhedonia/lack of positive affect is a core depressive symptom and a core negative symptom despite being completely absent in the CDSS and in the PANSS. Indeed, anhedonia is indeed probably the most specific depressive symptom, i.e., best differentiating between depression and other psychopathological states (including amongst others somatic complaints, anxiety, paranoia, schizophrenia, borderline features, etc. ….) (26). In a medically ill population, anhedonia was also shown to be the best screening symptom for depression, better than depressed mood or than fatigue (27). Anhedonia is also one of the five key constructs in negative symptoms (together with blunted affect, alogia, avolition and asociality) (13). The fact that anhedonia is absent in the CDSS and in the PANSS could well be because the authors found it difficult to disentangle the anhedonia as a depressive symptom and the anhedonia as a negative symptom. Addington indeed wrote that the “CDSS was meant to have only a single dimension with less sensitivity to overlap with other schizophrenia symptom dimensions” (8). Anhedonia indeed is a complex phenomenon including anticipatory and consummatory as well as sensory and social aspects. While in depression all aspects of the hedonic tone are impaired, the situation is more complex in schizophrenia where two anhedonia paradoxes have been described (28). The first is that schizophrenia patients seem to have a normal consummatory hedonic tone (liking, taking pleasure) while having an impaired anticipatory hedonic tone (wanting, seeking for, looking for); the second is that in contrast to patients with schizophrenia where the consummatory hedonic tone is normal, patients with prodromal phases or with schizotypy have an impaired hedonic tone (28). Since anhedonia is a core depressive symptom as well as a core negative symptom and since anhedonia seems to be intimately linked to suicidality, the complexity of the phenomenon should of course not result in deleting anhedonia from the assessment tools that are used in schizophrenia. It is therefore welcomed that newer scales like The Brief Negative Symptom Scale (BNSS) or the Clinical Assessment Interview for Negative symptoms (CAINS) do assess anhedonia and pleasure (29, 30).

Another important finding is that the network analysis, including both the PANSS and the CDSS items, clearly confirms the Mohr 5-factor model of the PANSS: the negative symptom factor, the hostility/excitement factor, the positive symptom factor, the cognitive factor and the mood factor are visually easily recognizable (7). This confirms a recently published study where this structure was also easily recognizable in both an acute patient population and in a population with predominant negative symptoms (6).

In conclusion, the present network analysis suggests that depressive symptoms (assessed with the CDSS or with the anxiety-depression subscale of the PANSS) and anxious symptoms (assessed with the anxiety-depression subscale of the PANSS) are the most central symptoms and that they are only poorly associated with negative symptoms in this population and hence are well distinguishable. Moreover, our network analysis shows clusters of symptoms that clearly support the Mohr 5 factor model. In the investigated population of stabilized patients with predominant negative symptoms depression (both assessed with CDSS and PANSS), anxiety, lack of judgment and insight and tension are the most central symptoms, suggesting that these symptoms should clinically well be taken into account.

## Data Availability Statement

The raw data supporting the conclusions of this article will be made available by the authors, without undue reservation.

## Ethics Statement

The clinical study protocol was approved by nine central and 37 local independent Ethics Committees in relation to the 66 sites that recruited at least one patient; the study was done in accordance with good clinical practice guidelines and the principles of the International Conference on Harmonization. All patients provided written informed consent. The patients/participants provided their written informed consent to participate in this study.

## Author Contributions

All authors listed have made a substantial, direct, and intellectual contribution to the work and approved it for publication.

## Conflict of Interest

The authors declare that the research was conducted in the absence of any commercial or financial relationships that could be construed as a potential conflict of interest.

## Publisher's Note

All claims expressed in this article are solely those of the authors and do not necessarily represent those of their affiliated organizations, or those of the publisher, the editors and the reviewers. Any product that may be evaluated in this article, or claim that may be made by its manufacturer, is not guaranteed or endorsed by the publisher.

## References

[1] Kahn RS Sommer IE Murray RM Meyer-Lindenberg A Weinberger DR Cannon TD. Schizophrenia. Nat Rev Dis Primers. (2015) 1:15067. 10.1038/nrdp.2015.67 27189524

[2] American Psychiatric Association. Diagnostic and Statistical Manual of Mental Disorders Fifth Edition (DSM-5). Arlington, VA: American Psychiatric Publishing (2013). 10.1176/appi.books.9780890425596

[3] Buckley PF Miller BJ Lehrer DS Castle DJ. Psychiatric comorbidities and schizophrenia. Schizophr Bull. (2009) 35:383–402. 10.1093/schbul/sbn135 19011234 PMC2659306

[4] Jiang T Nagy D Rosellini AJ Horváth-Puhó E Keyes KM Lash TL. The joint effects of depression and comorbid psychiatric disorders on suicide deaths: competing antagonism as an explanation for subadditivity. Epidemiology. (2021) 33:295–305. 10.1097/EDE.0000000000001449 34860728

[5] Tsai J Rosenheck RA. Psychiatric comorbidity among adults with schizophrenia: a latent class analysis. Psychiatry Res. (2013) 210:16–20. 10.1016/j.psychres.2013.05.013 23726869 PMC3800495

[6] Demyttenaere K Leenaerts N Acsai K Sebe B Laszlovszky I Barabássy A. Disentangling the symptoms of schizophrenia: network analysis in acute phase patients and in patients with predominant negative symptoms. Eur Psychiatry. (2021) 1–27. 10.1192/j.eurpsy.2021.2241 34641986 PMC8926909

[7] Mohr PE Cheng CM Claxton K Conley RR Feldman JJ Hargreaves WA. The heterogeneity of schizophrenia in disease states. Schizophr Res. (2004) 71:83–95. 10.1016/j.schres.2003.11.008 15374576

[8] Addington D Addington J Schissel B. A depression rating scale for schizophrenia. Schizophr Res. (1990) 3:247–51. 10.1016/0920-9964(90)90005-R 2278986

[9] Kay SR Fiszbein A Opler LA. The positive and negative syndrome scale (PANSS) for schizophrenia. Schizophr Bull. (1987) 13:261–76. 10.1093/schbul/13.2.261 3616518

[10] Hasan A Falkai P Wobrock T Lieberman J Glenthoj B Gattaz WF. World federation of societies of biological psychiaty (WFSBP) guidelines for biological treatment of schizophrenia. part 3: update 2015 management of special circumstances: depression, suicidality, substance use disorders and pregnancy and lactation. World J Biol Psychiatry. (2015) 16:142–70. 10.3109/15622975.2015.1009163 25822804

[11] Marder SR Galderisi S. The current conceptualization of negative symptoms in schizophrenia. World Psychiatry. (2017) 16:14–24. 10.1002/wps.20385 28127915 PMC5269507

[12] Leucht S Leucht C Huhn M Chaimani A Mavridis D Helfer B. Sixty years of placebo-controlled antipsychotic drug trials in acute schizophrenia: systematic review, bayesian meta-analysis, and meta-regression of efficacy predictors. Am J Psychiatry. (2017) 174:927–42. 10.1176/appi.ajp.2017.16121358 28541090

[13] Correll CU Schooler NR. Negative symptoms in schizophrenia: a review and clinical guide for recognition, assessment and treatment. Neuropsychiatr Dis Treat. (2020) 16:519–34. 10.2147/NDT.S225643 32110026 PMC7041437

[14] Borsboom D Cramer AO. Network analysis: an integrative approach to the structure of psychopathology. Annu Rev Clin Psychol. (2013) 9:91–121. 10.1146/annurev-clinpsy-050212-185608 23537483

[15] Dalege J Borsboom D van Harreveld F van der Maas HLJ. Network analysis on attitudes: a brief tutorial. Soc Psychol Personal Sci. (2017) 8:528–37. 10.1177/1948550617709827 28919944 PMC5582642

[16] Németh G Laszlovszky I Czobor P Szalai E Szatmári B Harsányi J. Cariprazine versus risperidone monotherapy for treatment of predominant negative symptoms in patients with schizophrenia: a randomised, double-blind, controlled trial. Lancet. (2017) 389:1103–13. 10.1016/S0140-6736(17)30060-0 28185672

[17] Simpson GM Angus JW. A rating scale for extrapyramidal side effects. Acta Psychiatr Scand. (1970) 212:11–19. 10.1111/j.1600-0447.1970.tb02066.x 4917967

[18] Epskamp S Borsboom D Fried EI. Estimating psychological networks and their 626 accuracy: a tutorial paper. Behav Res Methods. (2018) 50:195–212. 10.3758/s13428-017-0862-1 28342071 PMC5809547

[19] Law H Shryane N Bentall RP Morrison AP. Longitudinal predictors of subjective recovery in psychosis. Br J Psychiatry. (2016) 209:48–53. 10.1192/bjp.bp.114.158428 26585094

[20] Cassidy RM Yang F Kapczinski F Passos IC. Risk factors for suicidality in patients with schizophrenia: a systematic review, meta-analysis and meta-regression of 96 studies. Schizophr Bull. (2017) 44:787–97. 10.1093/schbul/sbx131 29036388 PMC6007264

[21] Babinkostova Z Stefanovski B. Forms of antipsychotic therapy: improved individual outcomes under personalised treatment of schizophrenia focused on depression. EPMA J. (2011) 2:391–402. 10.1007/s13167-011-0103-0 23199176 PMC3405399

[22] an der Heiden W Leber A Häfner H. Negative symptoms and their association with depressive symptoms in the long-term course of schizophrenia. Eur Arch Psychiatry Clin Neurosci. (2016) 266:387–96. 10.1007/s00406-016-0697-2 27107764

[23] American Psychiatric Association. Diagnostic and Statistical Manual of Mental Disorders Fourth Edition (DSM-IV). Arlington, VA: American Psychiatric Publishing (1994).

[24] Krynicki CR Upthegrove R Deakin JFW Barnes TRE. The relationship between negative symptoms and depression in schizophrenia: a systematic review. Acta Psychiatr Scand. (2018) 137:380–90. 10.1111/acps.12873 29532909

[25] Rahim T Rashid R. Comparison of depression symptoms between primary depression and secondary-to-schizophrenia depression. Int J Psychiatry Clin. (2017) 21:314–7. 10.1080/13651501.2017.1324036 28503978

[26] Olson TR Presniak MD MacGregor MW. Reevaluation positive affect in the center for epidemiologic studies-depression scale. Psychiatr Res. (2010) 178:545–9. 10.1016/j.psychres.2010.05.014 20538344

[27] Sibitz I Berger P Freidl M Topitz A Krautgartner M Spiegel W. ICD-10 of DSM-IV? anhedonia, fatigue and depressed mood as screening symptoms for diagnosing a current depressive episode in physically ill patients in general hospital. J Affect Disord. (2010) 126:245–51. 10.1016/j.jad.2010.03.023 20400184

[28] Strauss GP Cohen AS. The schizophrenia spectrum anhedonia paradox. World Psychiatry. (2018) 17:221–2. 10.1002/wps.20529 29856563 PMC5980495

[29] Kirkpatrick B Strauss GP Nguyen L Fischer BA Daniel DG Cienfuegos A. The brief negative symptom scale: psychometric properties. Schizophr Bull. (2011) 37:300–5. 10.1093/schbul/sbq059 20558531 PMC3044634

[30] Kring AM Gur RE Blanchar JJ Horan WP Reise SP. The clinical assessment interview for negative symptoms (CAINS): final development and validation. Am J Psychiatr. (2013) 170:165–72. 10.1176/appi.ajp.2012.12010109 23377637 PMC3785242

