# Supplementary figures and images for: Depressive Symptoms and PANSS Symptom Dimensions in Patients With Predominant Negative Symptom Schizophrenia: A Network Analysis
Source: Front Psychiatry. 2022 Apr 25;13:795866. doi: 10.3389/fpsyt.2022.795866 (PMC9081724; doi:10.3389/fpsyt.2022.795866)

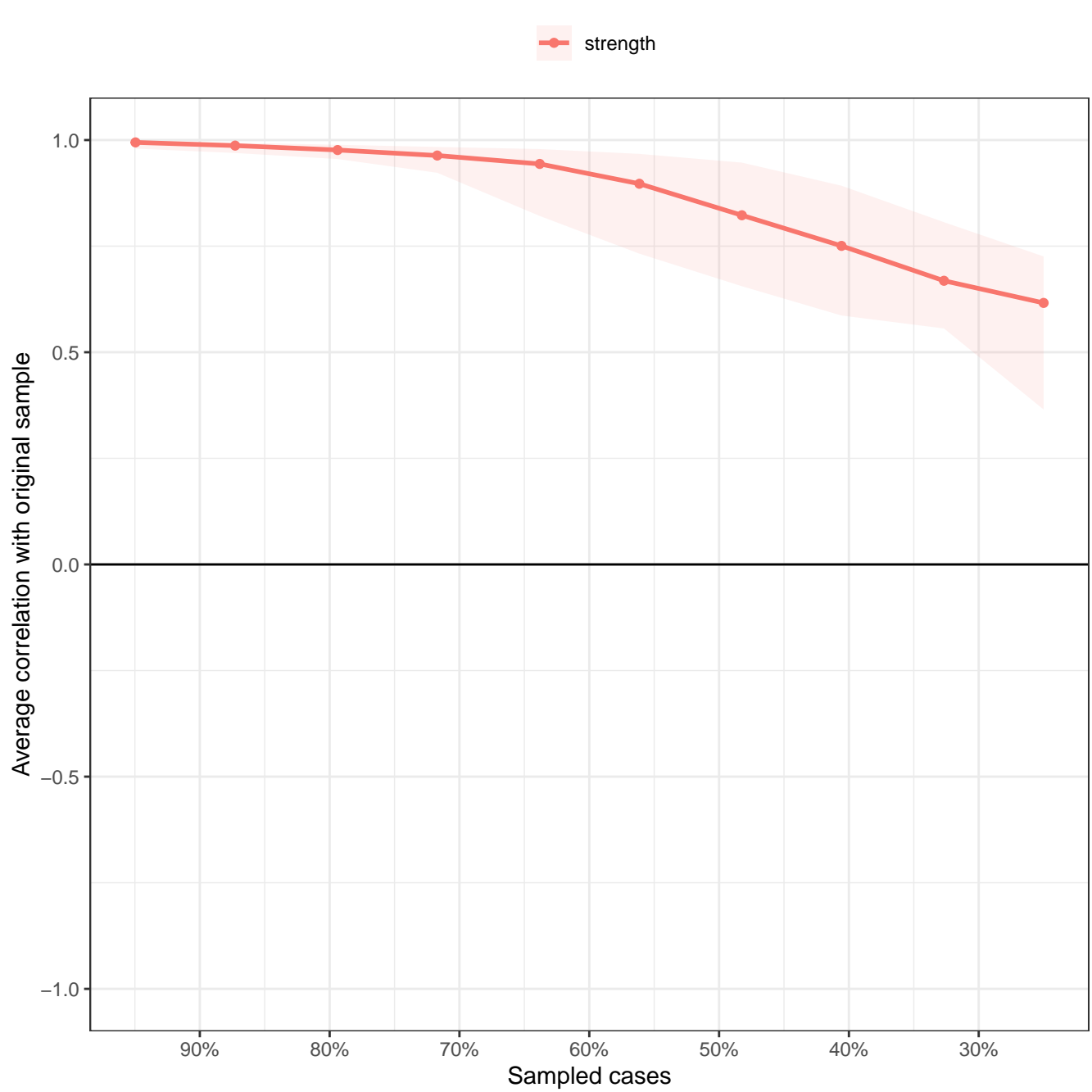

Supplement: Supplementary file 1 [file Data_Sheet_1.PDF]
